# Supplementary material for: LncMirNet: Predicting LncRNA–miRNA Interaction Based on Deep Learning of Ribonucleic Acid Sequences
Source: Molecules. 2020 Sep 23;25(19):4372. doi: 10.3390/molecules25194372 (PMC7583909; doi:10.3390/molecules25194372)
Supplement: Supplementary file 1 [file molecules-25-04372-s001.zip › Supplemental file/Supplemental Manual 1.pdf]

How to run LncMirNet?

There are four steps to run LncMirNet.

1) preparing lncRNA sequences and miRNA sequences with Fasta format.
2) using k-mer, CTD, doc2vec, and role2vec to calculate lncRNA sequences features and miRNA sequence features.
3) feed these features to the LncMirNet model.
4) the predicted results are listed in a result file with csv format.

Following the commands, you will get the predicted results.

create conda env and activate the env

```
conda create -n lnc_mir python=3.6
conda activate lnc_mir
```

install dependency

```
conda install numpy=1.18.5
conda install pandas=1.0.5
conda install networkx=2.4
conda install tensorflow-gpu=1.15
conda install keras=2.3.1
conda install scikit-learn=0.22
conda install biopython=1.77
pip install gensim==3.8.3
pip install tqdm==4.28.1
pip install texttable==1.5.0
pip install scipy==1.1.0
```

download source code

```
git clone https://github.com/abcair/LncMirNet.git
```

Modify file path

```
cd test
change lnc_path and mir_path in parameters.py file
```

```
lnc_path = "./lncRNA_test.fa"
mir_path = "./miRNA_test.fa"
result_path = "test_result.csv"
```

Run test

```
python test.py
```

Result

```
the predicted result will save in test_result.csv
```

In the test directory, we list a lncRNA.fasta demo file and a miRNA.fasta demo file. The predicted results are save in test_result.csv.
