# Supplementary figures and images for: LncMirNet: Predicting LncRNA–miRNA Interaction Based on Deep Learning of Ribonucleic Acid Sequences
Source: Molecules. 2020 Sep 23;25(19):4372. doi: 10.3390/molecules25194372 (PMC7583909; doi:10.3390/molecules25194372)

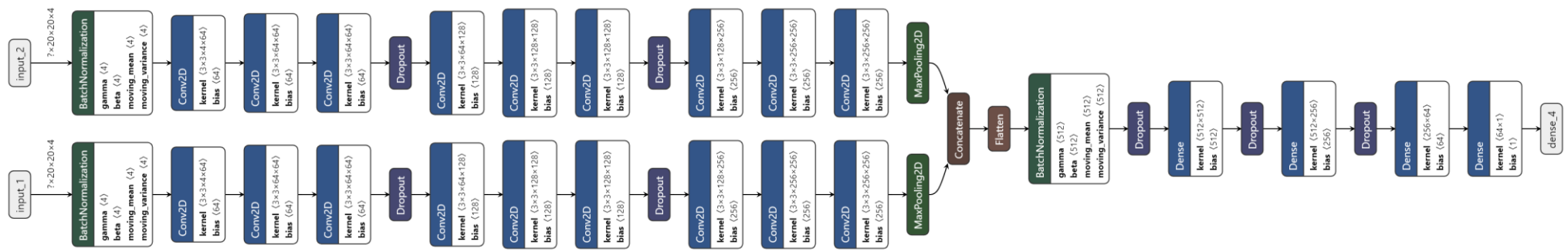

Supplementary Figure 1. The detailed struct of LncMirNet.

Supplement: Supplementary file 1 [file molecules-25-04372-s001.zip › Supplemental file/Supplementary Figure 1.pdf]
